# BEclear: Batch Effect Detection and Adjustment in DNA Methylation Data

**DOI:** 10.1371/journal.pone.0159921

**Published:** 2016-08-25

**Authors:** Ruslan Akulenko, Markus Merl, Volkhard Helms

**Affiliations:** 1 Center for Bioinformatics, Saarland University, Saarbruecken, Germany; 2 Graduate School for Computer Science, Saarland University, Saarbruecken, Germany; Beijing Cancer Hospital, CHINA

## Abstract

Batch effects describe non-natural variations of, for example, large-scale genomic data sets. If not corrected by suitable numerical algorithms, batch effects may seriously affect the analysis of these datasets. The novel array platform independent software tool BEclear enables researchers to identify those portions of the data that deviate statistically significant from the remaining data and to replace these portions by typical values reconstructed from neighboring data entries based on latent factor models. In contrast to other comparable methods that often use some sort of global normalization of the data, BEclear avoids changing the apparently unaffected parts of the data. We tested the performance of this approach on DNA methylation data for various tumor data sets taken from The Cancer Genome Atlas and compared the results to those obtained with the existing algorithms ComBat, Surrogate Variable Analysis, RUVm and Functional normalization. BEclear constantly performed at par with or better than these methods. BEclear is available as an R package at the Bioconductor project http://bioconductor.org/packages/release/bioc/html/BEclear.html.

## Introduction

The well-known batch effect describes a non-biological experimental variation that may result, in the case of genomic datasets, either from manufacturing problems of the diagnostic chips, from imprecisely conducted experiments, or simply from mislabeling of samples. Often it is very hard retrospectively to identify the exact cause of batch effects. In any case, batch effects often severely affect the large-scale automatic processing of genomic datasets [1]. It is therefore advisable to carefully check all data sets for the existence of batch effects and to adjust them if needed before any downstream analysis is performed.

The most straightforward way to avoid issues resulting from batch effects is to leave out from the analysis all seemingly affected batches of data or genes once they have been detected [2]. Yet, this is often not desirable since this reduces the coverage of the studied item. Another, certainly preferable, strategy is to always perform a sufficient number of replicate experiments. This would likely help in detecting problems such as mislabeling of data and may also reduce the effects of experimental mistakes if we assume that such mistakes occur at low frequencies. However, performing replicate experiments may still not overcome the problem of a systematic bias in the data if this is due, for example, to manufacturing problems affecting a certain charge of a diagnostic chip. Also, this is a costly option and is often not possible retrospectively.

In order to allow researchers to overcome problems resulting from batch effects, several algorithms for detecting [3] and dealing [4] with batch effects have been presented [5, 6, 7]. Typically, these approaches use some sort of global normalization approach. On the one hand, such approaches necessarily affect all data points in the complete dataset even though large portions of the data may be perfectly alright. On the other hand, normalization methods may not even be able to completely remove batch effect [8]. For example, even standard normalization techniques, which are part of accepted pipelines for transforming raw signal intensities for DNA methylation probes into calculated β-values mapped to the genome, might still be susceptible to batch effect S1 Fig).

Here, we present a novel approach for batch effect correction called BEclear. The numerical approach behind this is of general nature and may be applied to practically any sort of numerical data. For example, we have used it in several studies to replace ambiguous values detected with a DNA microarray from *S*. *aureus* samples [9, 10, 11]. The BEclear software presented here was developed for processing epigenetic data for cytosine methylation in DNA samples. Therefore, we will discuss the workflow and principles of the method on the example of DNA methylation. First, the tool applies the well-known Kolmogorov-Smirnov test to identify samples and genes that deviate significantly from the remaining data. Second, the software exploits a matrix approximation scheme termed latent factor models that is well-established e.g. in the field of recovering images from partial or corrupted data [12, 13]. In this way, BEclear replaces the batch affected entries by typical values observed for this gene in other, non-affected samples. We critically compared the performance of the method to the existing tools Combat, SVA, Functional Normalization and RUVm. We emphasize that, in contrast to these other methods, the BEclear correction is applied solely to the affected genes, leaving the data for other members of the sample unchanged.

## Materials and Methods

### Analyzed data sets

To illustrate the performance of BEclear and for comparing it to other tools, DNA methylation data for tumor and adjacent normal tissue for several cancer types were downloaded from The Cancer Genome Atlas (TCGA) data portal [14]. In this study we considered array-based DNA methylation data either at the so-called level 1 (raw signal intensities of probes for each participant's sample obtained by the HumanMethylation450 chip [15]) or at the level 3 (calculated β-values mapped to the genome). Our batch effect detection and correction method BEclear was established using level 3 data for breast invasive carcinoma (BRCA) with 745 tumor and 96 adjacent normal samples and then applied to other cancer types as well as to level 1 data.

### Preprocessing of data

In a similar way as done in [2], data from TCGA were locally stored in a MySQL database and then pre-processed. Tumor and adjacent normal data were considered separately to avoid batch effects resulting from the data mixture. As a first cleaning step, we removed all entries with missing β-values or missing gene names as well as entries with indistinct gene names. In the next step, we kept only those probes that overlap with the promoter regions of genes. For this we used annotations from the Eukaryotic Promoter Database EPDnew [16] as a reference for the location of transcription start sites (TSS) for every human gene. Thus, HumanMethylation450 DNA methylation probes were mapped to EPDnew data by gene name and chromosome, and only probes lying within 2000 bp up- or downstream of the annotated TSS (depending on the strand direction) were kept for further analysis. After this step, some genes were still represented by multiple probes in a single sample file. When working with level-3 data, we assigned the mean β-value of all its respective entries to those genes. Finally, this gave 11154 gene– β-value pairs in tumor matched data and 11213 in adjacent normal.

### Batch effect detection and correction method BEclear

#### Detection of batch affected samples

We used the batch identifier from the TCGA data portal to assign every single sample to its respective batch. In order to find out whether the data are affected by batch effects at the sample level, several standard visualization approaches were applied separately to tumor and adjacent normal samples, namely box plots, density plots, heat map together with hierarchical clustering, and principal component analysis (PCA).

#### Detection of batch effected genes (BE-genes)

Genes within a batch that are likely affected by batch effects were identified based on statistical analysis of batch medians. First, we compared the distribution of every gene in one batch to its distribution in all other batches using the nonparametric Kolmogorov-Smirnov (KS) test [17–20]. P-values returned by KS-test were corrected by False Discovery Rate, FDR [21].Second, to consider only biologically relevant differences in methylation levels, we identified the absolute difference between the median of all β-values within a batch for a specific gene and the respective median of the same gene in all other batches. We term this the median difference. Those genes that had a FDR-corrected significance p-value below 0.01(KS-test) (test 1) and had a median difference larger than 0.05 (test 2) were considered as batch effected genes in a specific batch. Importantly, the list of BE-genes differs for each batch.

#### Batch effect scoring (BE-score) and correction

After identifying single BE-genes we scored the severeness of batch effect in single batches by a weighting-scheme where we grouped each BE-gene into various bins. Each bin stands for a certain difference level between the median in this batch and the median of the other batches. Bins standing for larger differences then are weighted more strongly. Precisely, the BE-score was computed as:

$$BEscore=\frac{\sum_{i\in mdif_{cat}}\left(N_{BEgenes_{i}}\cdot w_{i}\right)}{N}$$ (1)

Here *N* is the total number of genes in a current batch, *mdif*_*cat*_ is the category of median differences, $N_{BEgenes_{i}}$ is the number of BE-genes belonging to the i-th *mdif* category and *w*_*i*_ is the weight for the respective *mdif* category. Weights were assigned in the following way:

1. if *mdif* < 0.05, then weight = 0;
2. if 0.05 ≤ *mdif <* 0.1 weight = 1;
3. if *m* × 0.1 ≤ *mdif* < (*m* + 1) × 0.1,.*m* belongs to *N*^*+*^

This scoring scheme considers the number of BE-genes in the batch as well as the magnitude of the deviation of the medians of BE-genes in one batch compared to all other batches.

Based on the BE-scores of all batches, we then identifiedusingtheDixon test from the "*outliers*" R package [22] which batches have BE-scores that deviate significantly from the BE-scores of the other batches (S1 Table). All such batches were flagged as batch effected batches and all BE-gene entries in these affected batches were replaced by predicted values. Latent Factor Models (LFM) based on matrix factorization [12, 13]. The main advantage of this method is the ability to incorporate both gene and sample preferences by taking into account the values of neighbor entries when predicting a missing value. Assuming that the dataset is represented by the *m* × *n* matrix *D* with rank *r*, LFM iteratively constructs an *m* × *r* matrix *L* and an *r* × *n* matrix *R* such that matrix multiplication [*LR*]_*ij*_ approximately equals to *D*_*ij*_ for every unaffected entry *A*. The Gradient descent optimization algorithm was used to minimize the global loss i.e. the difference between [*LR*] and *D*. Once it converges, entries that were not batch effected were preserved in the completed matrix *D*_*comp*_ = [*LR*] from the original data matrix *D*, so that the algorithm replaced only the matrix entries for BE-genes in the affected batches. In case if some of the predicted entries lie below 0 or above 1, they were assigned values of 0 and 1, respectively.

#### Method validation

During the detection of single batch effected genes in adjacent normal BRCA data we tested different values for *mdif*, p-value and different p-value adjustment methods. For *mdif* values larger than 0.1, only few genes (from 103 to 1465 BE-genes) were detected as BE-genes. After removing them, the batch effect was still visually observed. On the other hand, when *mdif* was set to values in the order of 0.01, more than 82% of all genes were detected as BE-genes. Thus, *mdif* in the order of 0.1 is a reasonable value. Next, we found that different thresholds for the p-value did not have noticeable effects on the results. With a p-value = 0.05, BEclear identified 5990 BE-genes and 5032 BE-genes for p-value = 0.001. Furthermore, three different p-value adjustment methods (FDR, Hommel and Bonferroni) yielded similar numbers of BE-genes of around 5500 genes.

We note that due to the usage of the Kolmogorov—Smirnov test, BEclear might not detect batch effect in batches containing fewer than 5 samples unless the batch effect is very strong as in the case of Kidney renal clear cell carcinoma KIRC, where the KS-test was compensated by large *mdif* values for BE-genes S7B Fig).Thus, we recommend a minimum number of 5 samples for application of BEclear.

#### Computational aspects

The matrix completion method was assessed in terms of overall accuracy and prediction time when applied to DNA methylation data. For testing purposes we used again the BRCA adjacent normal dataset. As a measure of accuracy, we computed the average absolute deviation between known and predicted entries (beta-values) of the matrix. Due to the fact that BEclear found 5.8% entries to be affected, testing was performed on 6% of additional randomly selected entries. Generally, the time needed to perform LFM prediction grows exponentially with the size of the data. For the BRCA dataset studied here (11213 genes in 96 samples), this task could be infeasible without separating the initial matrix into blocks of data and running LFM independently for every block. This approach also enables parallel execution on multi-core processors, what leads to significant savings in computation time.

We also analyzed how the size of the block of data to which LFM was applied affected the prediction accuracy. This parameter was varied from 10 to 250 in increments of 5. In all cases LFM yielded a similar accuracy that differed at most by 0.02 (S5 Fig).Note that in case when the size of the block of data is too large, this significantly affects the computation time without bringing an improvement in accuracy. On the other hand, a very small block size might not incorporate gene preference since there might be some large batch with batch effect. Also, the block could contain some inner part of that batch.

## Results and Discussion

### Batch effect detection and correction for BRCA data

**Fig 1:**
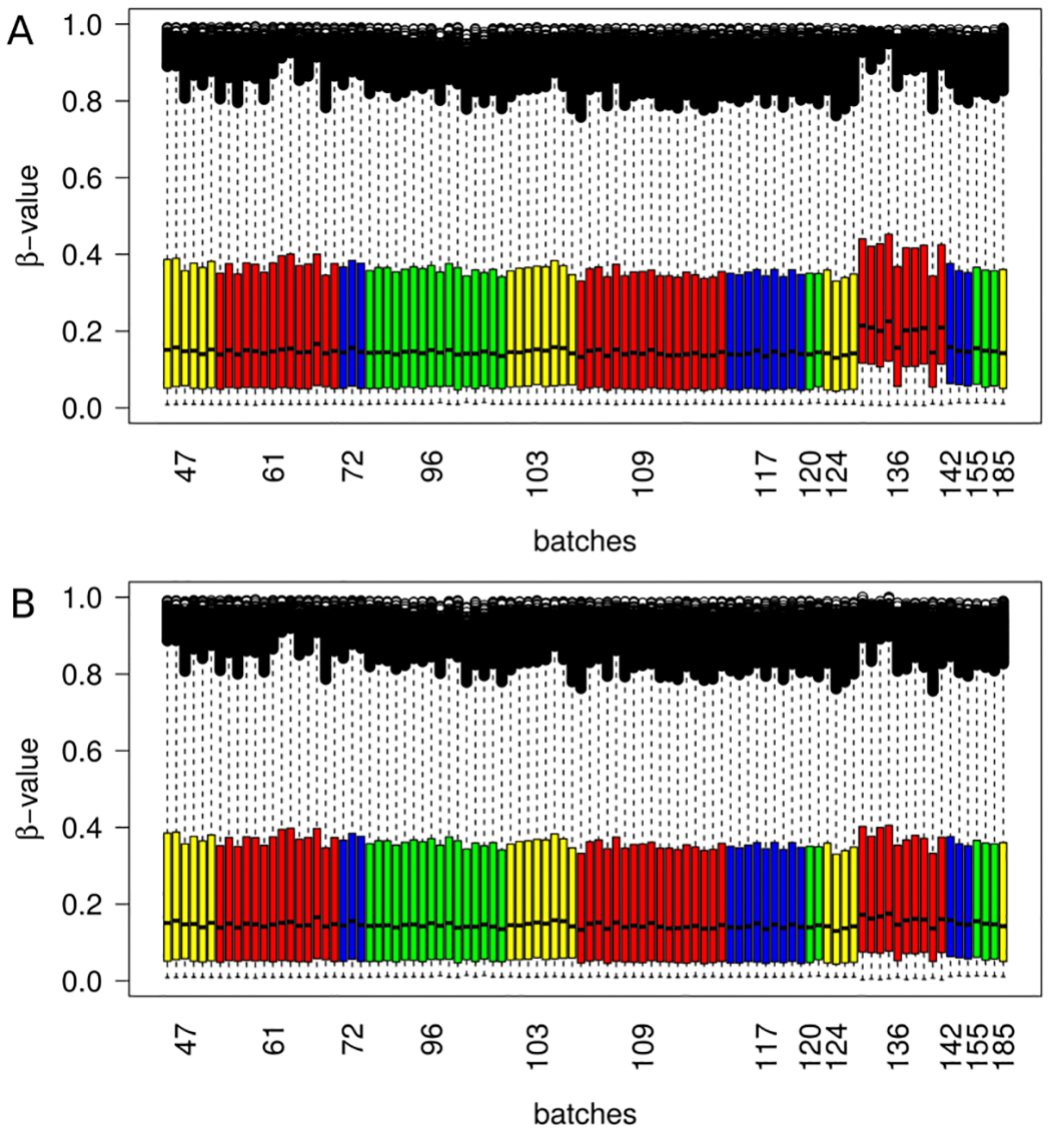
Box plots of adjacent normal breast cancer samples from TCGA (level 3 data—calculated β-values mapped to the genome), per sample level (96 samples). **A.** before batch effect adjustment. The p-value < 0.001 for BE-score of batch 136 (Dixon test) **B.** after applying BEclear method.

We start by illustrating the performance of BEclear using DNA methylation data for breast cancer (BRCA) samples downloaded from the TCGA portal [14]. For simplicity, we start with the analysis of level-3 data where methylation values are aggregated into one value per gene. Below, we present an analogous analysis of breast cancer level-1 data with BEclear. In that case, the notion of a batch-effected gene (that is used in the following) should be replaced by the notion of a batch-effected probe. S2 Fig shows box plots representing the distribution of β-values (proportion of methylatedCpG nucleotides ranging from 0 to 1) for all genes in BRCA samples both on a per-sample and a per-batch basis. These plots illustrate clearly that, in batch 136, the distribution of β-values of genes is shifted to larger values than in the other batches. The per sample plot (Fig 1A) shows that the difference in batch 136 is not due to only one sample but exists in all but two samples from this batch. Also the tumor data (S2B Fig) of batch 136 show a general increase of β-values. However, the difference is not as large as in the adjacent normal data, as seen in the per-sample plot, where only 15 out of 27 samples behave differently compared to other batches. This batch effect in adjacent normal data is also well apparent in the PCA, heatmap, and density plots (S3 Fig). Clearly, most samples from batch 136 tend to cluster together (S3A and S3B Fig) and the density of this batch is less sharp and shifted compared to other batches (S3C Fig).

This result observed by visual inspection was also confirmed by the BEclear method. For adjacent normal data we identified the number of batch affected genes (BE-genes) in every batch belonging to the respective *mdif* categories for the difference of median values (S2 Table). For example, the distribution of β-values for the SPINK2 gene in batch 136 (S4 Fig) is statistically significantly different from its distribution in all other batches (KS-test p-value = 9.41·e-6). This dataset is clearly affected by a strong batch effect in batch 136 since approximately 47% of all genes in this batch differ from their median β-value in the other batches by more than 0.05 (BE-score = 0.605; p-value < 0.001 Dixon test for BE scores). The batch effect in batch 136 in BRCA tumor data is not as drastic as in adjacent normal data but still has a BE-score of 0.19 (S3 Table; p-value < 0.001 Dixon test for BE scores).BEclear adjusted the methylation values of 6079 genes in13 batches in adjacent normal data and of 3587 genes in 31 batches in tumor data (Fig 1B) what successfully reduced the batch effect.

### Batch effect scoring of other tumor types

Additionally we assessed six further cancer types that are well represented at the TCGA portal in terms of batch effects (S1 Table). BEclear identified one further minor batch effect in tumor samples of Kidney renal clear cell carcinoma (KIRC) with p-value <0.001 (Dixon test). A similar finding was recently reported in [5].As for BRCA data, TCGA provides many KIRC batches but batch 32 is represented by only 2 samples (S7A Fig). Even though this batch doesn't contain many BE-genes, the median difference *mdif* of those genes is quite large (S7B Fig), yielding a BE-score of 0.185.

### Comparison of BEclear against existing BE correction methods on real data

Next, we compared BEclear against several well established methods for batch effect correction. We note that the individual methods require different pre-knowledge about the data. BEclear, Combat and FunNorm require knowledge of the batches, whereas SVA and RUVm require that two classes of data exist (for example, tumor and normal samples). ComBat [4] is a part of the Surrogate Variable Analysis package [6] in R [23]. It uses an empirical Bayes framework based on a location (mean)/ scale (variance) model. The method adjusts the data so that all batches have similar values of means and variances in all batches. Since DNA methylation data generally do not follow a normal distribution, we opted for the nonparametric version of ComBat to correct BRCA data. Before running the batch effect adjustment, batches 185 and 93 were excluded from normal and tumor data, respectively, because ComBat is not able to handle batches just containing a single sample.

We separately corrected adjacent normal (S8A Fig) and tumor (S8B Fig) data using ComBat. The tool was obviously able to remove the observed batch effect in batch 136 by equalizing upper quartiles, medians and lower quartiles for every box in normal data. In contrast to the adjacent normal data, the variation between the range of the boxes in tumor data is mostly maintained compared to the original data whereas the formerly outstanding batch 136 is obviously corrected and boxes are shifted to a similar level compared to the other batches. Inspection of the number of BE-genes remaining after BE correction showed that both ComBat and BEclear were able to remove batch effect and had a similar performance (S9 Fig).

In the course of this comparison we noticed the following differences between ComBat and BEclear. As mentioned before, ComBat cannot handle batches that only contain a single sample and assumes as default that the data follow a normal distribution. As is typical for normalization methods, ComBat adjusts all entries in the dataset even though not all of them are affected by batch effect. Especially in the tumor data, which inherently contains more variation, we speculate that the strict adjustment of the data by Combat might diminish biological variation. In contrast, BEclear leaves all unaffected parts of the data as is and only replaces the entries of batch effected genes in certain batches by the predicted entry based on the gene and samples preference. One artifact is that ComBat produces many values above 1 and below 0, whereas β -values must be inside the interval [0;1] by definition (S10 Fig). For tumor data after BE correction, ComBat produced 261 values above 1 and 6529 below 0. Some of these values exceed the allowed interval by more than 0.15. In contrast, BEclear yielded only 32 for each case and the maximum deviation was 0.06. In the case of adjacent normal data only few such cases were observed (above 1: ComBat 0, BEclear 3; below 0: Combat 37, BEclear 0). It should be mentioned that ComBat was originally designed to handle batch effects in gene expression data where the value range is not restricted to stay between 0 and 1. In cases, where most of the genes are unmethylated, ComBat will shift the data strongly towards 0 resulting in many entries lying below 0 (S10B Fig). Such problems arise with BEclear much less often. We finally eliminated this problem in BEclear by cutting values at 0 and 1.

**Fig 2:**
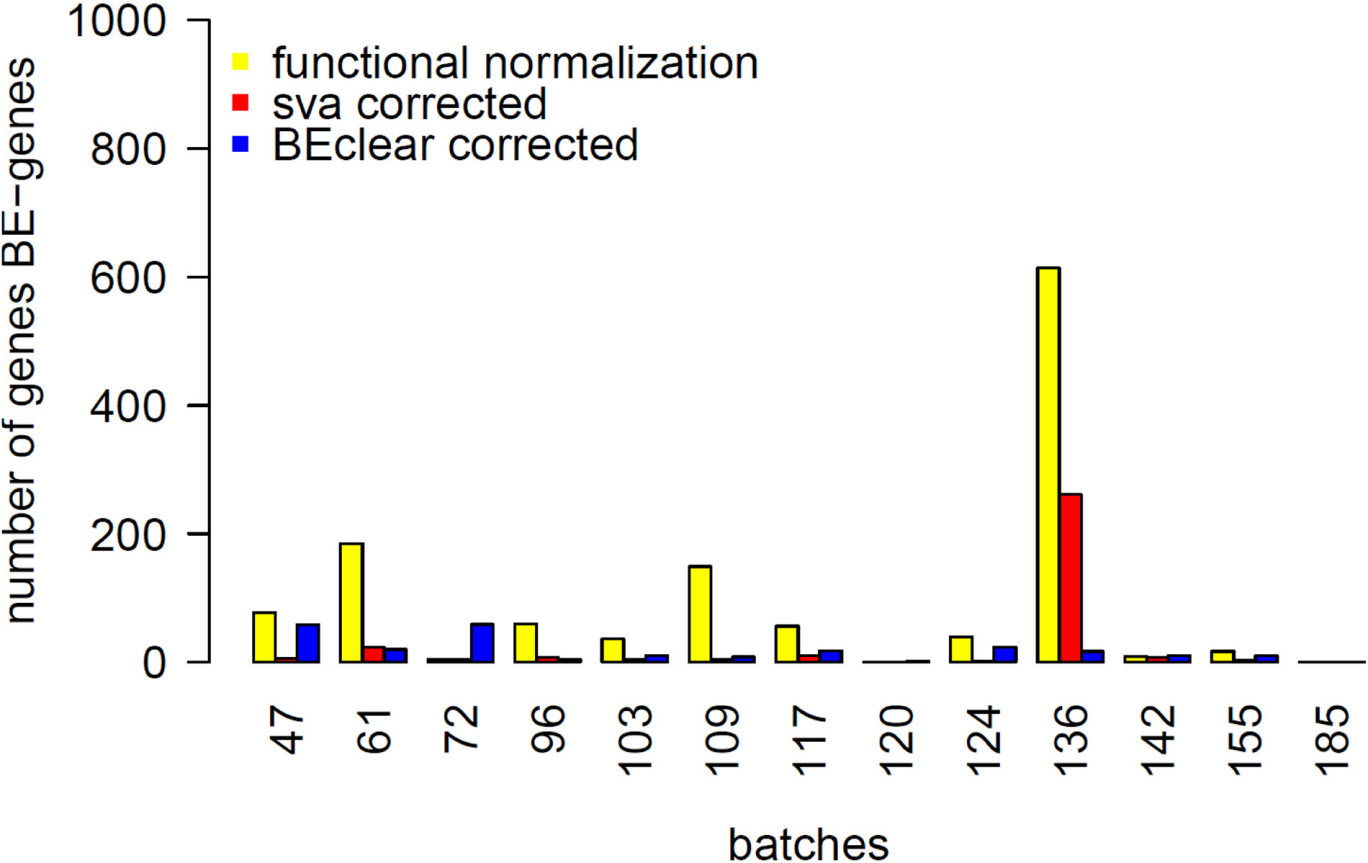
Comparison of BEclear, SVA and Functional normalization (minfi package) with respect to the number of BE-genes still remaining after the correction of adjacent normal BRCA data. Batch affected genes are defined as genes with (1) median difference above 5% of β-value distribution and (2) showing a statistically significant difference in this batch compared to all other batches with (p-value ≤ 0.01) according to the Kolmogorov-Smirnov test.

Then, we compared BEclear against Surrogate Variable Analysis (SVA). When applying *sva* and *fsva R* functions [6] to level 3 adjacent normal and tumor BRCA data, we noticed that SVA was able to remove batch effect to a large extent still preserving variation in the data, in distinction to ComBat. Indeed, the adjustment done by SVA appears less effective or too cautious than that of BEclear. Evidence for this provided by Fig 2 showing the number of genes that stilled showed significant differences between batches (KS-test) after batch effect adjustment. Here, adjustment by BEclear gave far less BE-genes than SVA.

Finally, we compared BEclear to the recently presented method Functional normalization that was designed specifically for the 450k methylation array [5, 24]. At first glance, Functional normalization was indeed able to remove batch effect (S11A Fig). However, the density of batch 136, the most affected group of samples, still differs from the density of other batches (KS-test p-value = 4.03·e-4, S11B Fig). After batch effect correction, Functional normalization still gave 1128 BE-genes (of which 755 belong to batch 136, the most affected batch) whereas BEclear only left 223 BE-genes (20 from batch 136), respectively (Fig 2). Besides, almost half (1353 out of 3804) of all human housekeeping genes[25] are affected by batch effect in the original data (S12A Fig) what leads to an increase of the methylation level in the most affected batch 136. Since the promoter regions of housekeeping genes should be generally unmethylated, we studied their behavior in the adjacent normal BRCA data before batch effect correction and after applying BEclear or functional normalization (S12 Fig). Especially focusing on those 1353 batch affected housekeeping genes clearly showed that batch 136 is still shifted slightly upwards after functional normalization what is not the case for BEclear where all bars have approximately equal first, third quartiles and median.

### Benchmarking of BEclear against existing BE correction methods using simulated data

**Fig 3:**
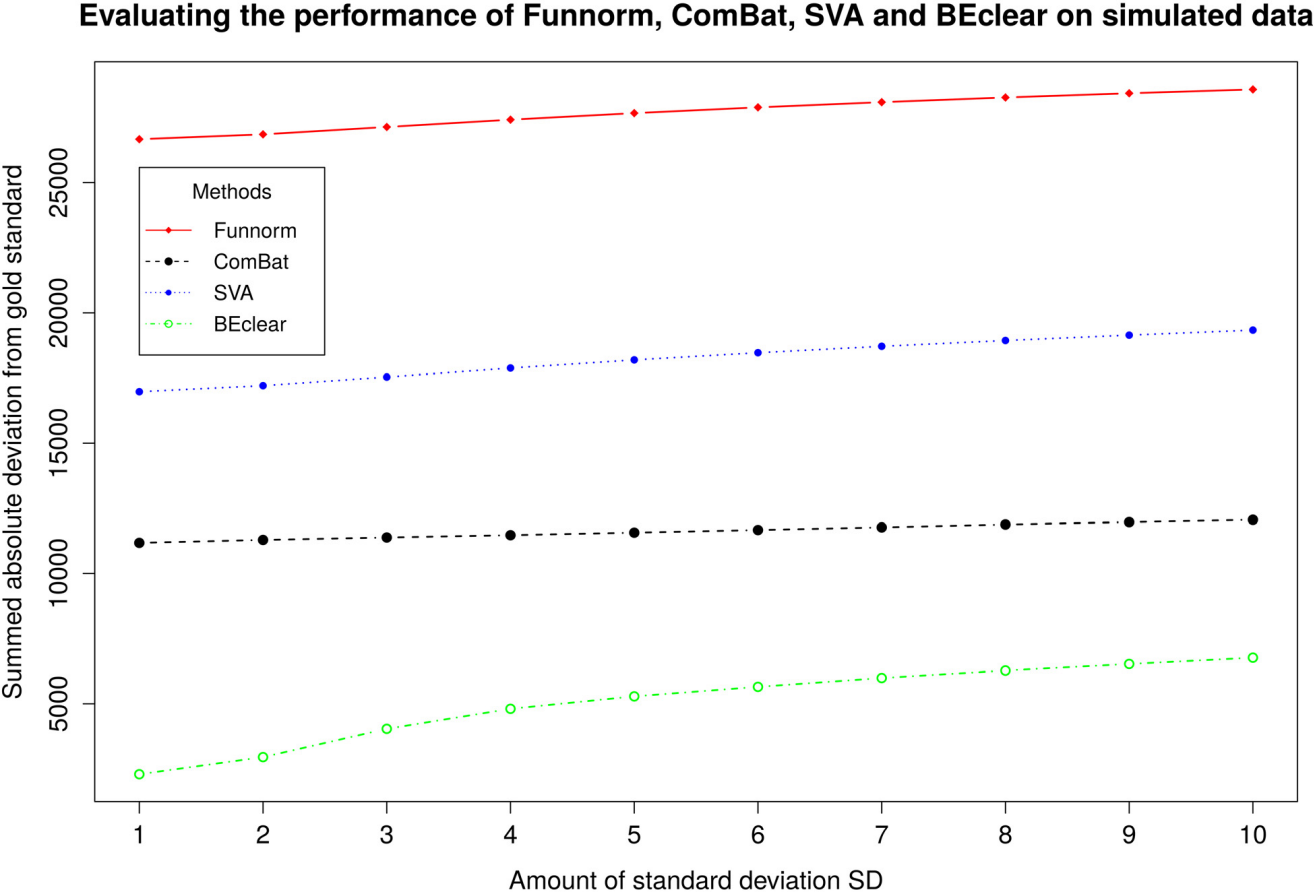
Comparison of BEclear, SVA, ComBat and Functional normalization using simulated batch effect. On the x-axis, we quantify the magnitude of the introduced batch effect perturbation in terms of multiples of the standard deviation of the data. As a measure of performance, the y-axis shows the total absolute difference of level 1 β-value between gold standard data and corrected entries for 8000 probes in 13 batches.

For a systematic comparison of batch effect correction methods, we generated synthetic data sets with “known” batch effects as described in [26]. First, we determined the standard deviation of the methylation value of each promoter probe in level 1 adjacent normal samples (samples belonging to batch 136 were excluded due to the existing batch effect).Then we randomly selected 8000 promoter probes (approximately 10% of all promoter probes present on the chip) and increased the methylation values of 4000 of these promoter probes by a specified multiple of their specific standard deviation plus a noise term [27]. The original probe values before introducing the synthetic batch effect were considered as our gold standard. Finally, every method except RUVm [7] was applied to the simulated data and the values of the adjusted probes were compared to the golden standard. This procedure was performed for different multiples of standard deviation, ranging from 1 to 10 (Fig 3).

**Fig 4:**
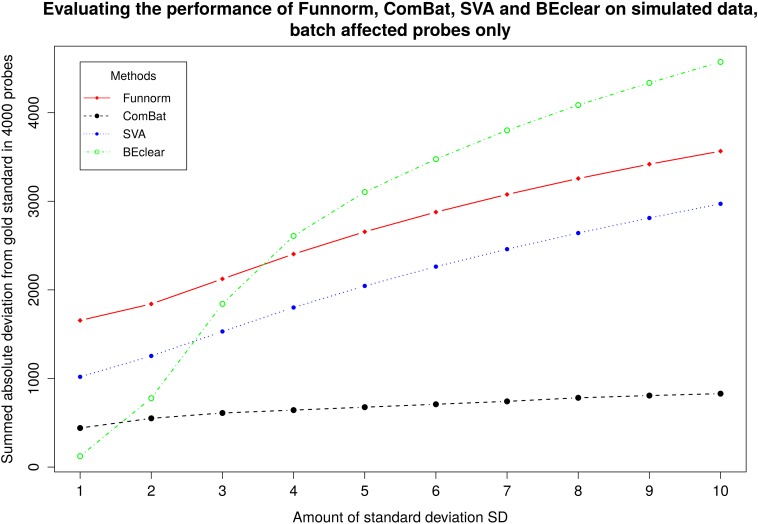
Comparison of BEclear, SVA, ComBat, and Functional normalization using simulated batch effect (compare Fig 3). As a measure of performance we used the total absolute difference between gold standard data and corrected entries for 4000 batch affected probes in batch 136.

When considering all 8000 probes including the 4000 shifted probes,BEclear gave a much smaller total deviation between the methylation values of BEclear-adjusted probes and the original gold standard value. We believe that this is the case because BEclear adjusts only batch affected entries and keeps the other entries at their original values. When focusing on the values of the 4000 probes that were synthetically shifted (batch affected), then the performance of BEclear relative to the other methods depended on the magnitude of the introduced batch effect. For small perturbations (of 1 SD or 2 SD) that are typical magnitudes in real situations, BEclear performed comparably well as Combat and better than SVA and FunNorm (Fig 4). For perturbations larger than 3 SD, BEclear gave larger total deviations than the other methods for the affected probes.When we repeated the same experiment with only 1000 affected probes, then BEclear had a similar behavior as SVA and FunNorm (S13 Fig). These can be explained by considering that BEclear bases its predictions on the values of neighboring cells. Thus, the larger the fraction of corrected (i.e. not batch effected) probes is, the more accurate are the values of the predicted entries. Thus, the expected magnitude of batch effects and the expected fraction of affected probes are crucial factors in selecting the appropriate method for correcting the batch effect.

**Fig 5:**
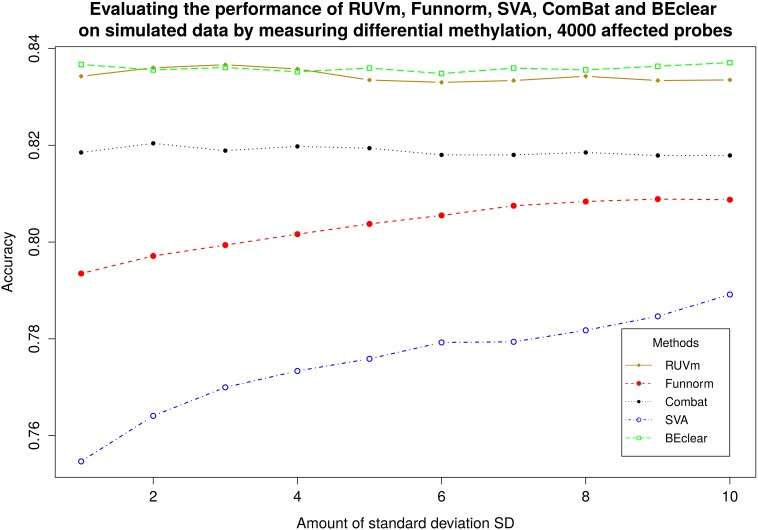
Comparison of RUVm, BEclear, SVA, ComBat, and Functional normalization using simulated batch effect. For all methods the list of differentially methylated genes (DMG) was obtained and then compared to the list of DMG for gold standard data. Here batch affect was introduced to 4000 probes.

For benchmarking against the RUVm method we considered the identities of differentially methylated genes in breast tumor samples vs. normal samples. As gold standard reference, we used the list of differentially methylated probes identified in the unaffected data using the limma package [28]. Then, we designed a synthetic batch effect in a similar fashion as in Fig 4 and applied BEclear, RUVm, FunNorm, ComBat, and SVA to this data. Then, again we identified differentially methylated genes in this BE-adjusted data with limma and compared the results to the original data. Fig 5 shows the accuracy defined as(TP + TN) / (TP + TN + FP + FN) for the difference BE-adjustment methods. BEclear yielded a similar accuracy as RUVm and both methods were more accurate compared to all others. Again, repeating this experiment for 1000 affected probes slightly increased the accuracy of BEclear compared to RUVm (S14 Fig).

### Co-methylation and differential methylation analysis

Co-methylation analysis of gene pairs was performed in the same manner as in our previous work [2] on BRCA data before and after applying BEclear, in order to investigate the impact of batch effect on the amount of artifacts. Since the data are already preprocessed and contain promoter region methylation, only pairwise Pearson correlation and 3 step filtering needed to be computed. As the number of tumor samples significantly exceeds the number of adjacent normal samples, only samples coming from the same participants were considered for the combined dataset. Matching them by TCGA barcodes resulted in 190 samples all together. In our previous study, we excluded all batch affected genes from the analysis [2]. This had the undesired effect of removing about one quarter of all genes. Here, this filtering step could be avoided. S4 Table lists the number of pairs of genes with correlation higher than 0.75 or lower than -0.75 for the original data and for the BE-adjusted data. Clearly, batch effects are responsible not only for generating false associations between genes with respect to their methylation levels in different samples, but also for losing a large portion of expected relationships. This behavior doesn't depend on the data type and can be observed in tumor, adjacent normal and combined samples.

Finally, differential methylation analysis between tumor and normal samples was carried out by applying KS-test [17–20] and Significance analysis of microarrays SAM [29, 30] for 190 combined adjacent normal and tumor samples. We emphasize that the point of this analysis is not to advocate BEclear as a novel or better method to perform differential methylation analysis. Instead, this section is meant to illustrate the problems resulting in differential methylation analysis if batch effects are not corrected. The KS-test returned the list of genes whose distribution in normal samples differs from the distribution in tumor samples with p-value below 0.01. To verify this list, SAM was applied independently and only genes returned by both methods were considered for further analysis. In this way two lists of differentially methylated genes were generated—one list for data without batch effect correction and another list for data after applying BEclear. These lists contain 6147 and 6672 genes, respectively. 616 genes of the second list were not contained in the first list meaning that they were only identified to be differentially methylated after batch effect adjusting. This latter group of genes contained many genes which are known to play an important role during cancer development or even have been associated with breast cancer before: *NRG4*, *TUBB*, *LPL*, *BRD2*, *MYB*, *RAP2C*, *SIRT7*, *MAZ*, *HRAS*, *TXN*, *PPM1D*, *TP53I3*, *PARK7*, *TP63* [31–45]. Importantly, these genes would not have been identified to be differentially methylated based on the original data.

Finally, we tested our BEclear method on raw adjacent normal data from the HumanMethylation450 chip without initial preprocessing. In this case, it was searching for batch affected CpGs instead of BE-genes and included both types of probes—from the promoter region and other regions of the gene. It was able to detect and adjust batch effect in the same batch 136 with BE-score = 1.47 (Dixon test p-value < 0.001). Thus we recommend to use BEclear after applying dedicated normalization methods in order to assess batch effect; and in the case of its presence—to adjust it.

### Recommended use of BEclear

In this paragraph, we summarize the results from comparing BEclear to the alternative tools SVA, Combat, Functional normalization and RUVm.(1) The first and main advantage of BEclear over all other methods is that BEclear preserves the original measurements to the highest possible extent. Only entries for the batch affected genes in the distorted samples are adjusted by BEclear; all other data points of non-affected genes and also of the batch affected genes in the non-distorted samples are kept at their original values. In our view, this strategy is beneficial whenever the researcher cares about the absolute values of the data entries, not only about relative trends between them. In contrast, all other methods use some sort of normalization and modify the values of essentially all data points. (2) Only ComBat and BEclear can handle data where all samples belong to one class. This helps, for example, in adjusting tumor data that is not accompanied by corresponding adjacent normal samples. Furthermore, BEclear can handle single sample batches whereas this option is not available for ComBat. (3)In contrast to Functional normalization and RUVm, the three methods BEclear, SVA and ComBat are platform independent and can be applied to different levels of data ranging from raw signals to mapped and aggregated β-values. Yet, we showed that SVA and ComBatdid not reach the same level of performance in differential methylation analysis as BEclear and RUVm. (4) RUVm can only correct batch effects in the context of differential methylation analysis. This is not the case for BEclear, SVA and ComBat. (5) BEclear outperformed all other methods on the simulated data in terms of accuracy for batch effects ofless than two standard deviations in magnitude (which is typically the case).

BEclear applies rigorous statistics to detect whether or not individual batches in the input data are affected by batch effects. The appropriate measure to decide this is the BEscore value computed by the program. Depending on the purpose of the experiment, BEclear can be either used alone or combined with other post processing methods.

## Conclusions

We have compared BEclear against other well established methods for batch effect adjustment. Depending on the metrics used and the strength of batch effect, BEclear either outperformed other methods or performed comparably well. As the other methods all use some form of normalization, they affect all data entries. Such normalization approaches may be most appropriate to correct for technical variations (or errors) where all probes on an array are affected in a more or less similar way. In contrast, BEclear adjusts only those portions of the data that were identified to differ significantly from the other batches. This strategy may be useful, for example, to process data from diagnostic chips showing some inhomogeneity or ambiguity in certain areas/entries. Thus, we suggest BEclear as a novel method to control batch effects in the data remaining after application of standard normalization techniques.

## Supporting Information

S1 Fig Per sample boxplot of adjacent normal BRCA data from TCGA, level 1 data. This stands for DNA methylation raw signal intensities of probes for each participant's sample. Batch effect is clearly present in batch 136 since the distribution of β-values in these samples significantly deviates from the other samples. This illustrates that the background correction technique applied by the *methylumi* package when processing level 1 data into level 3 data did not remove the batch effect in the batch 136.
(PNG)

S2 Fig Box plots of breast cancer samples from TCGA (level 3 data). A. Adjacent normal samples per batch level (13 batches). B. Tumor samples, per batch level (32 batches). C. Subset of tumor samples for batch 136 and surrounding batches, per sample level. All these plots illustrate clearly that batch 136 is affected by batch effect in both tumor and adjacent normal samples.
(TIFF)

S3 Fig Visual inspection of batch effect in adjacent normal breast invasive carcinoma data from TCGA (level 3 data). A. The heatmap demonstrates that all but two samples from batch 136 form a cluster that splits off from the other samples at the top of the hierarchy. B. Plotting the first two Principle Components and projecting samples on them clearly distinguishes samples from batch 136 from the rest. C. The density plot of every batch shows that the β-values in batch 136 have a different distribution than in the other batches.
(TIFF)

S4 Fig Per batch boxplot of the β-values for gene *SPINK2* in adjacent normal breast invasive carcinoma data from TCGA (level 3). For this gene, we identified the largest difference of 0.428 between the median of batch 136 and the median of the other batches.
(PNG)

S5 Fig Accuracy assessment of the Latent Factor Model. Here, we investigated the impact of the block size on the overall accuracy of LFM matrix completion. Four parameters were computed: mean, median, minimal and maximum difference between actual and predicted β- value entries. The size of the block of the data, to which LFM was applied, was varied from 10 to 250. Larger block sizes increase the frequency of large β-value differences (green curve). Overall, LFM shows good prediction accuracy in a wide range of data block sizes whereby the median of the difference remains in the range of 0.01 and the mean stays around 0.02.
(PNG)

S6 Fig Results of batch effect correction of breast cancer data from TCGA using BEclear. A. Per batch boxplot of corrected adjacent normal data. B. Per batch boxplot of corrected tumor data. C. Density plot and D. PCA plot of corrected adjacent normal data. In the per batch boxplot of corrected normal data (S6A Fig) batch 136 does not stand out explicitly anymore. This is also confirmed by the per sample boxplot (Fig 1B from main text). Even though the tumor dataset had a smaller batch effect than adjacent normal samples, it was successfully adjusted and now the bar corresponding to batch 136 is in a similar range compared to other batches (S6B Fig). Additionally, S6C and S6D Fig confirm the positive effect of BEclear on normal data. The corrected data of batch 136 is now positioned next to all other batches. However, it is also apparent that a certain variation between samples remains since BEclear adjusted only the methylation values of BE-genes.
(TIFF)

S7 Fig DNA methylation data for kidney renal clear cell carcinoma tumor samples, KIRC, from TCGA. A. Per sample boxplot. Batch 32, which contains only two samples, has a batch effect score equal to 0.185 signaling that its data should be corrected. B. Number of genes belonging to different categories of median differences (mdif) between genes in the current batch and the same gene in all other batches (as described in section 2.3.3. “Batch effect scoring” in the main text).
(TIFF)

S8 Fig Correction of batch effect in BRCA data from TCGA using the tool ComBat. The previously observed batch effect in batch 136 was corrected both in A. adjacent normal and B. tumor data.
(TIFF)

S9 Fig Comparison of original TCGA data, data adjusted by ComBat, and data adjusted by our correction method BEclear. Shown are the number of batch effected genes in single batches from A. BRCA adjacent normal and B. BRCA tumor data.
(TIFF)

S10 Fig Comparison of the tools ComBat and BEclear with respect to the number of wrongly predicted entries with incorrect β-values below 0 or above 1. Note that BEclear sets these values eventually to 0 and 1. A. Boxplot of entries with values larger than one in the breast cancer tumor data from TCGA adjusted either by ComBat or by BEclear. B. The same as in A showing the number of values below 0. C. Boxplot of values below 0 in adjacent normal data after correction by ComBat.
(TIFF)

S11 Fig Results of batch effect adjustment of breast cancer adjacent normal data from TCGA using the tool Functional normalization. A. Per sample boxplot B. Density plot. Functional normalization was able to adjust the batch effect equally well as BEclear since S11A Fig looks very similar to what was obtained after BEclear correction (S6B Fig).
(TIFF)

S12 Fig Boxplots of 1353 batch affected housekeeping genes in adjacent normal breast invasive carcinoma data from TCGAA. before any batch effect adjustment B. after functional normalization C. after batch effect correction with BEclear. The most affected batch is marked in red.
(TIFF)

S13 Fig Comparison of BEclear, SVA, ComBat and Functional normalization using simulated batch effect (compare section 3.4 in the main text and Fig 4. In contrast to the main section, here only 1000 samples were perturbed). As a measure of performance we used the total absolute difference of the β-values between gold standard data and corrected entries for 1000 batch affected probes (out of 8000 probes).
(PNG)

S14 Fig Comparison of RUVm, BEclear, SVA, ComBat and Functional normalization using simulated batch effect (compare Fig 5 in the main text. Here, only 1000 probes were perturbed instead of 4000). For all methods the list of differentially methylated genes (DMG) was obtained and then compared to the list of DMG for gold standard data. Here batch affect was introduced to 1000 probes (out of 8000). The x-axis indicates for the magnitude of the introduced batch effect.
(PNG)

S1 Table BE scoring of DNA methylation data for 7 different cancer types from the TCGA portal. Cancer types and batches which were identified to have a batch effect are marked in bold. This table contains the description of cancer types, batch identifiers obtained from the TCGA portal and the batch effect score (see Eq (1) main text). Only those batches with BE-score over 0.01 are listed here since, generally, every batch has some extremely small non-zero BE-scores. This is due to some variation in a few genes and only in rare cases the BE-score for a batch is exactly zero. All the batches belonging to LUSC have a BE-score in the range of (0; 0.01) because not more than 97 genes in a single batch behave differently compared to other batches. The Dixon test yielded p-values for testing whether the BE-score of one of the batches differs significantly from the others in the same cancer type. Note that Dixon test is applied to a set of batches for one condition, not to a single batch. Hence, the reported p-value belongs to the respective set of batches. Note also that the Dixon test should not be considered alone but with BE-score threshold = 0.1, since it is prone to finding significant deviations of BE-scores if they are close to 0, as in the case for UCEC (adjacent normal samples) and THCA (adjacent normal and tumor samples) data.
(DOCX)

S2 Table BE scoring of batches in BRCA adjacent normal data from TCGA. The median difference counts the number of genes for which the median DNA methylation in this batch differs from its median in all other batches by a value falling into the respective intervals specified at the top. The BEscore is computed according to Eq (1) in the main text.
(DOCX)

S3 Table BE scoring of batches in BRCA tumor data from TCGA (similar to S1 Table). (DOCX)

S4 Table Results of co-methylation analysis. Listed are the number of highly co-methylated pairs of genes (Pearson correlation higher than 0.75 or lower than -0.75) for three different types of data after batch effect adjustment with BEclear and before.
(DOCX)
