# Supplementary figures and images for: BEclear: Batch Effect Detection and Adjustment in DNA Methylation Data
Source: PLoS One. 2016 Aug 25;11(8):e0159921. doi: 10.1371/journal.pone.0159921 (PMC4999208; doi:10.1371/journal.pone.0159921)

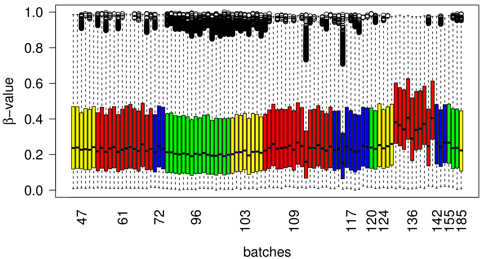

Supplement: S1 Fig — This stands for DNA methylation raw signal intensities of probes for each participant's sample. Batch effect is clearly present in batch 136 since the distribution of β-values in these samples significantly deviates from the other samples. This illustrates that the background correction technique applied by the methylumi package when processing level 1 data into level 3 data did not remove the batch effect in the batch 136. (PNG) [file pone.0159921.s001.png]

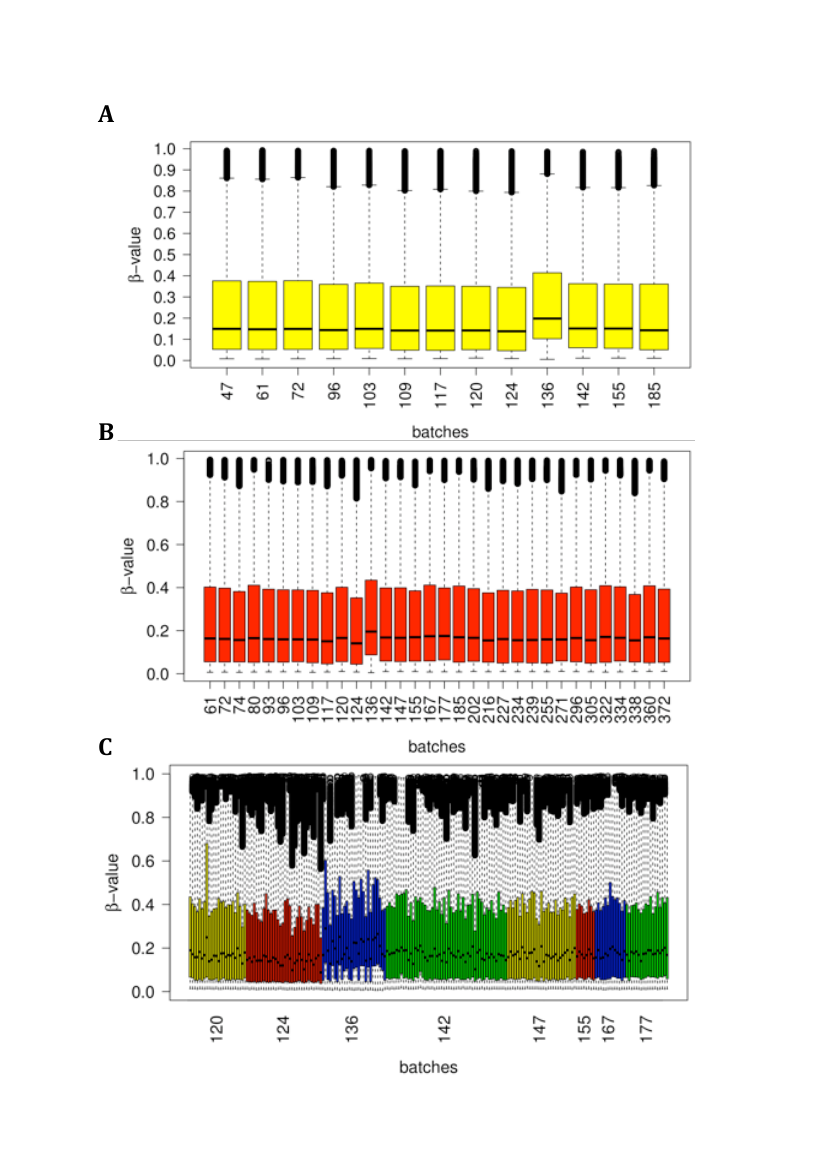

Supplement: S2 Fig — A. Adjacent normal samples per batch level (13 batches). B. Tumor samples, per batch level (32 batches). C. Subset of tumor samples for batch 136 and surrounding batches, per sample level. All these plots illustrate clearly that batch 136 is affected by batch effect in both tumor and adjacent normal samples. (TIFF) [file pone.0159921.s002.tiff]

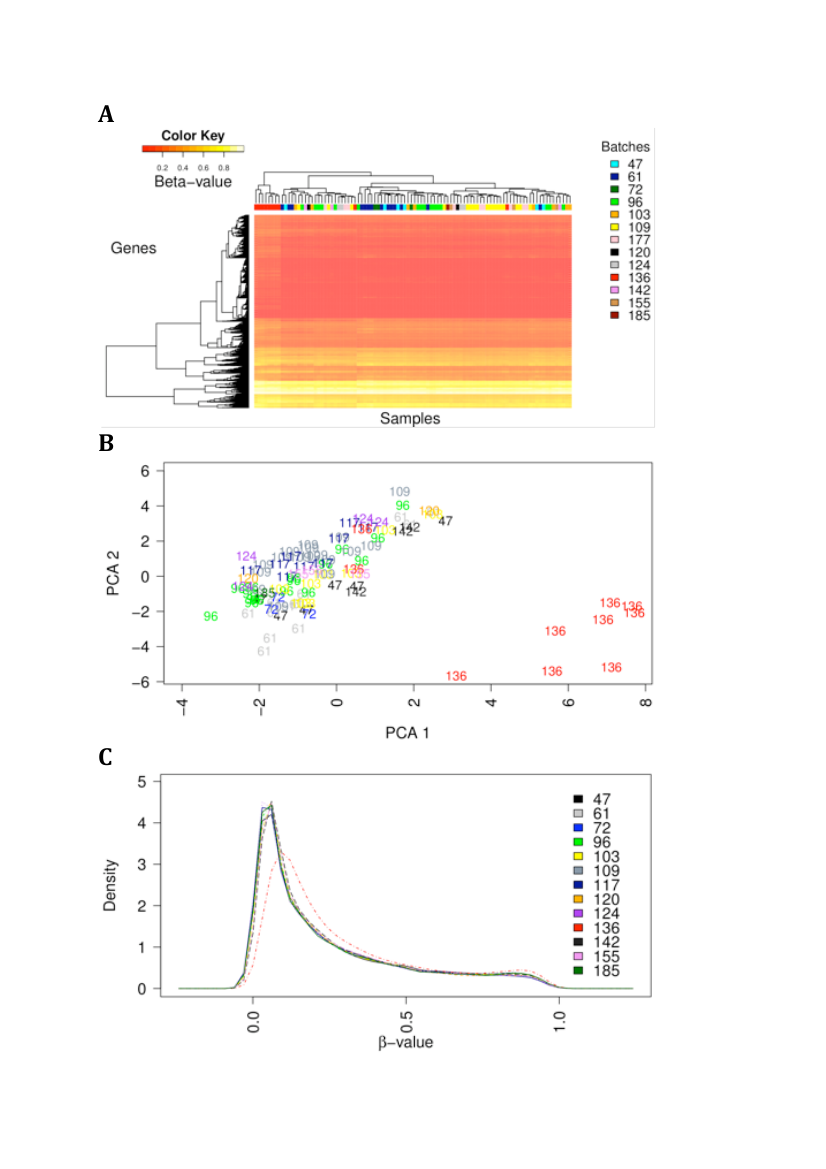

Supplement: S3 Fig — A. The heatmap demonstrates that all but two samples from batch 136 form a cluster that splits off from the other samples at the top of the hierarchy. B. Plotting the first two Principle Components and projecting samples on them clearly distinguishes samples from batch 136 from the rest. C. The density plot of every batch shows that the β-values in batch 136 have a different distribution than in the other batches. (TIFF) [file pone.0159921.s003.tiff]

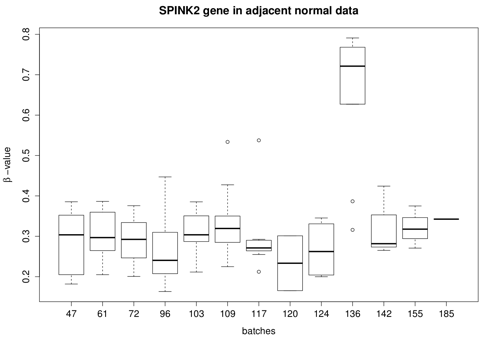

Supplement: S4 Fig — For this gene, we identified the largest difference of 0.428 between the median of batch 136 and the median of the other batches. (PNG) [file pone.0159921.s004.png]

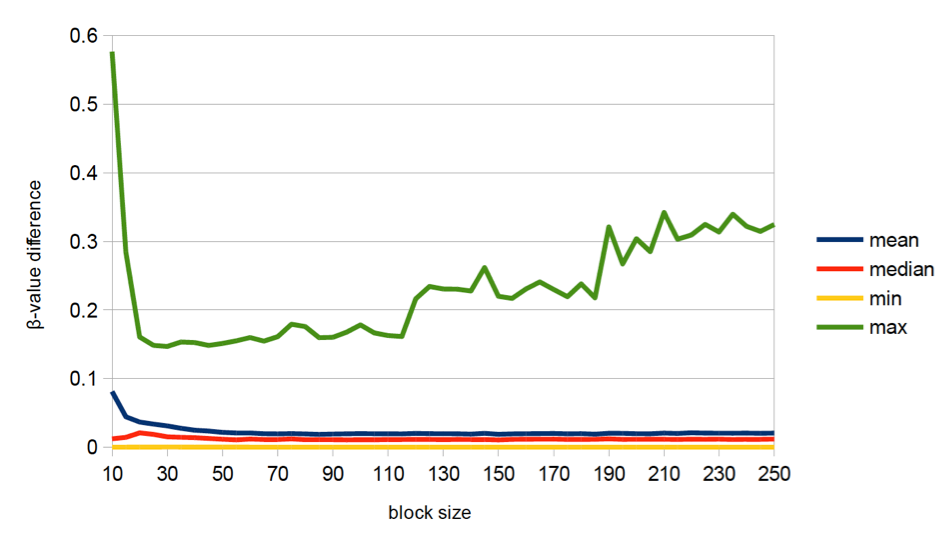

Supplement: S5 Fig — Here, we investigated the impact of the block size on the overall accuracy of LFM matrix completion. Four parameters were computed: mean, median, minimal and maximum difference between actual and predicted β- value entries. The size of the block of the data, to which LFM was applied, was varied from 10 to 250. Larger block sizes increase the frequency of large β-value differences (green curve). Overall, LFM shows good prediction accuracy in a wide range of data block sizes whereby the median of the difference remains in the range of 0.01 and the mean stays around 0.02. (PNG) [file pone.0159921.s005.png]

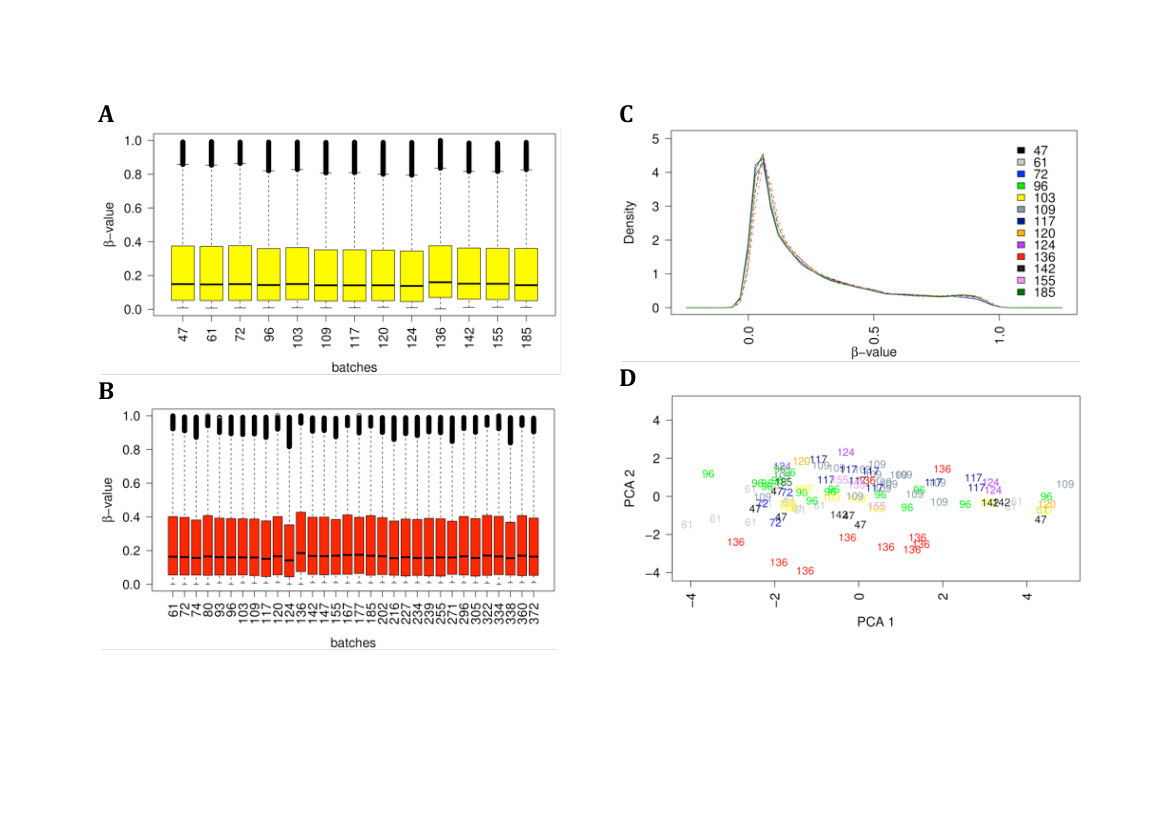

Supplement: S6 Fig — A. Per batch boxplot of corrected adjacent normal data. B. Per batch boxplot of corrected tumor data. C. Density plot and D. PCA plot of corrected adjacent normal data. In the per batch boxplot of corrected normal data (S6A Fig) batch 136 does not stand out explicitly anymore. This is also confirmed by the per sample boxplot (Fig 1B from main text). Even though the tumor dataset had a smaller batch effect than adjacent normal samples, it was successfully adjusted and now the bar corresponding to batch 136 is in a similar range compared to other batches (S6B Fig). Additionally, S6C and S6D Fig confirm the positive effect of BEclear on normal data. The corrected data of batch 136 is now positioned next to all other batches. However, it is also apparent that a certain variation between samples remains since BEclear adjusted only the methylation values of BE-genes. (TIFF) [file pone.0159921.s006.tiff]

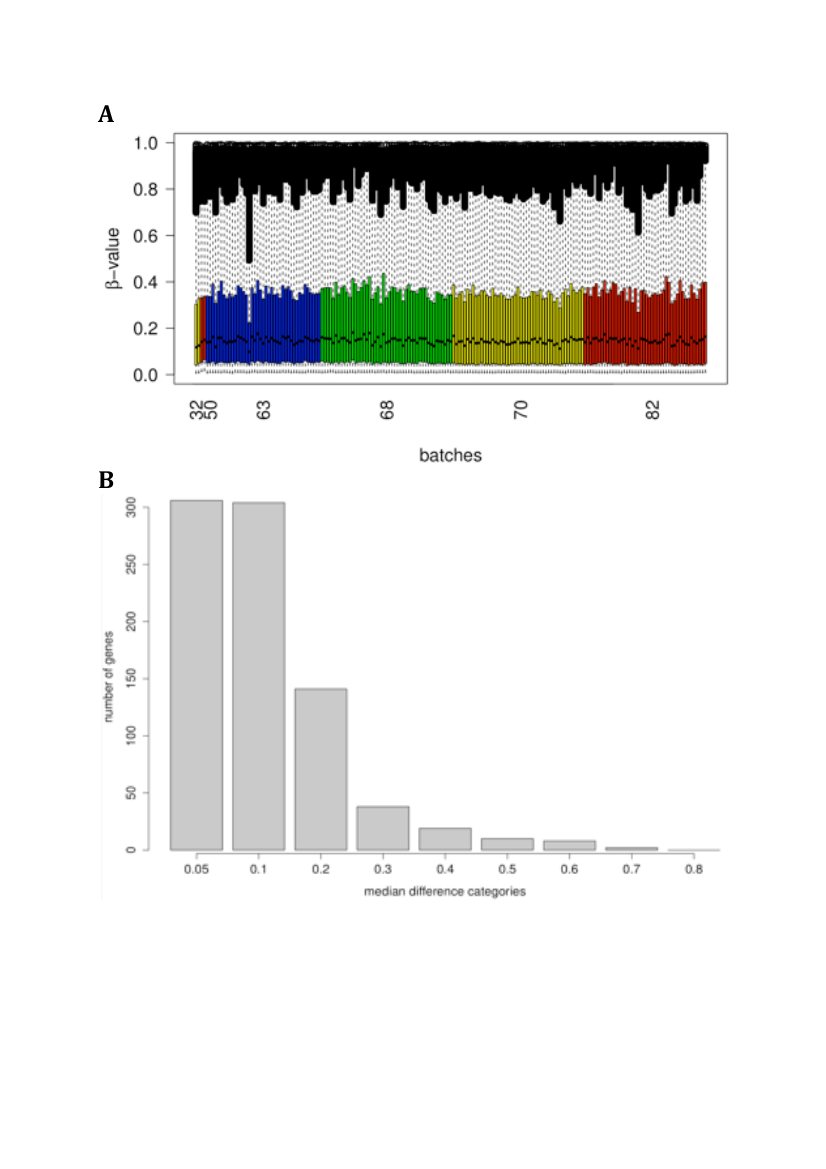

Supplement: S7 Fig — A. Per sample boxplot. Batch 32, which contains only two samples, has a batch effect score equal to 0.185 signaling that its data should be corrected. B. Number of genes belonging to different categories of median differences (mdif) between genes in the current batch and the same gene in all other batches (as described in section 2.3.3. “Batch effect scoring” in the main text). (TIFF) [file pone.0159921.s007.tiff]

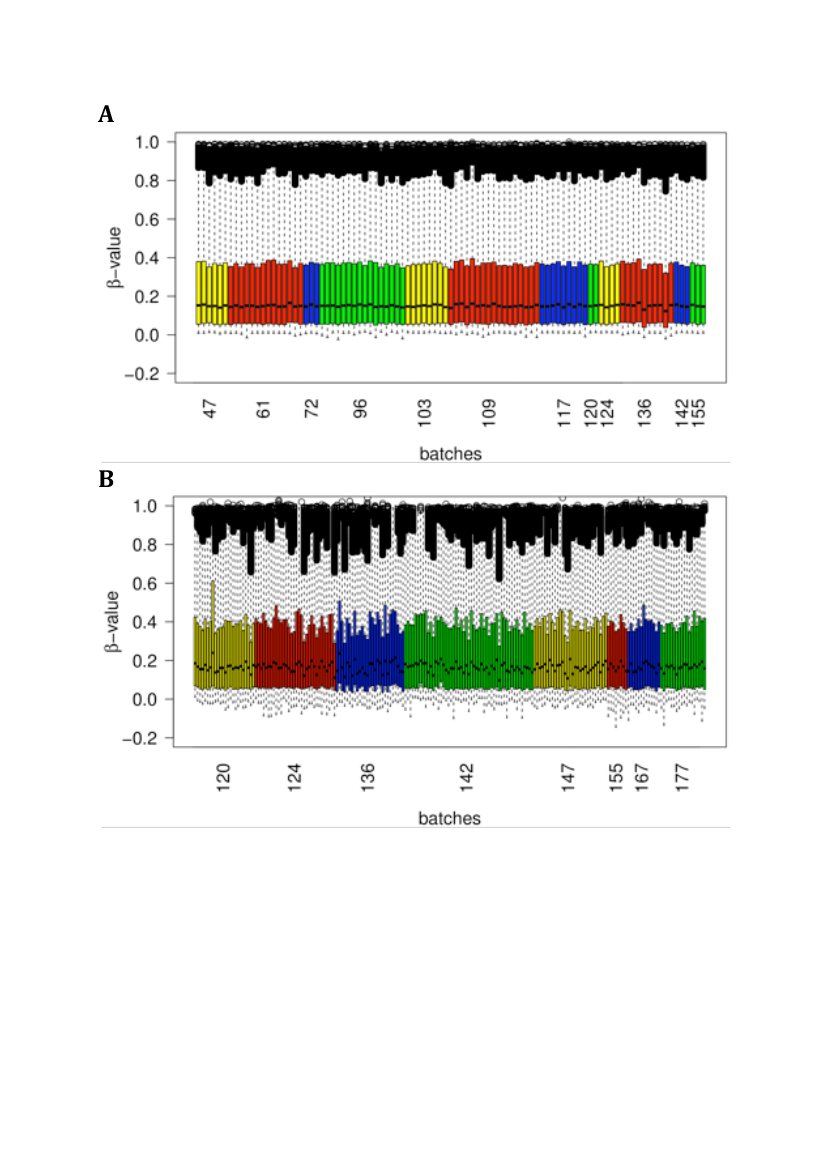

Supplement: S8 Fig — The previously observed batch effect in batch 136 was corrected both in A. adjacent normal and B. tumor data. (TIFF) [file pone.0159921.s008.tiff]

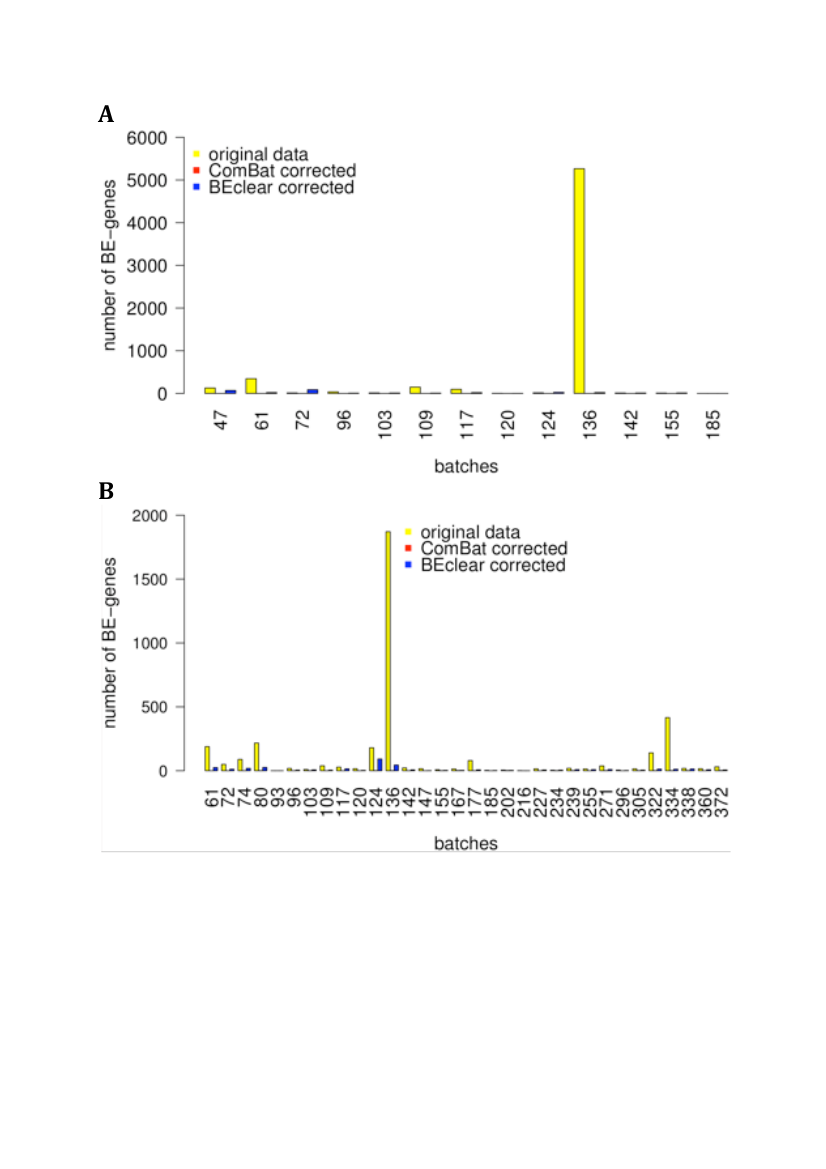

Supplement: S9 Fig — Shown are the number of batch effected genes in single batches from A. BRCA adjacent normal and B. BRCA tumor data. (TIFF) [file pone.0159921.s009.tiff]

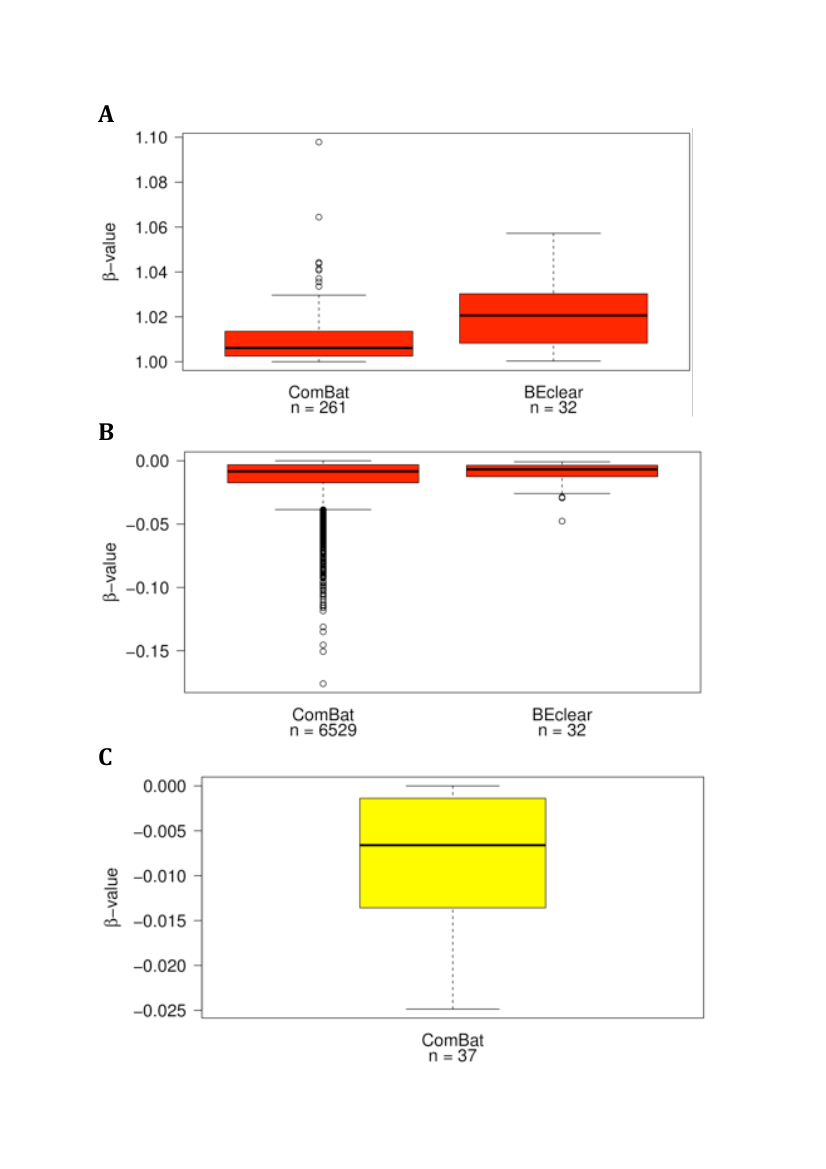

Supplement: S10 Fig — Note that BEclear sets these values eventually to 0 and 1. A. Boxplot of entries with values larger than one in the breast cancer tumor data from TCGA adjusted either by ComBat or by BEclear. B. The same as in A showing the number of values below 0. C. Boxplot of values below 0 in adjacent normal data after correction by ComBat. (TIFF) [file pone.0159921.s010.tiff]

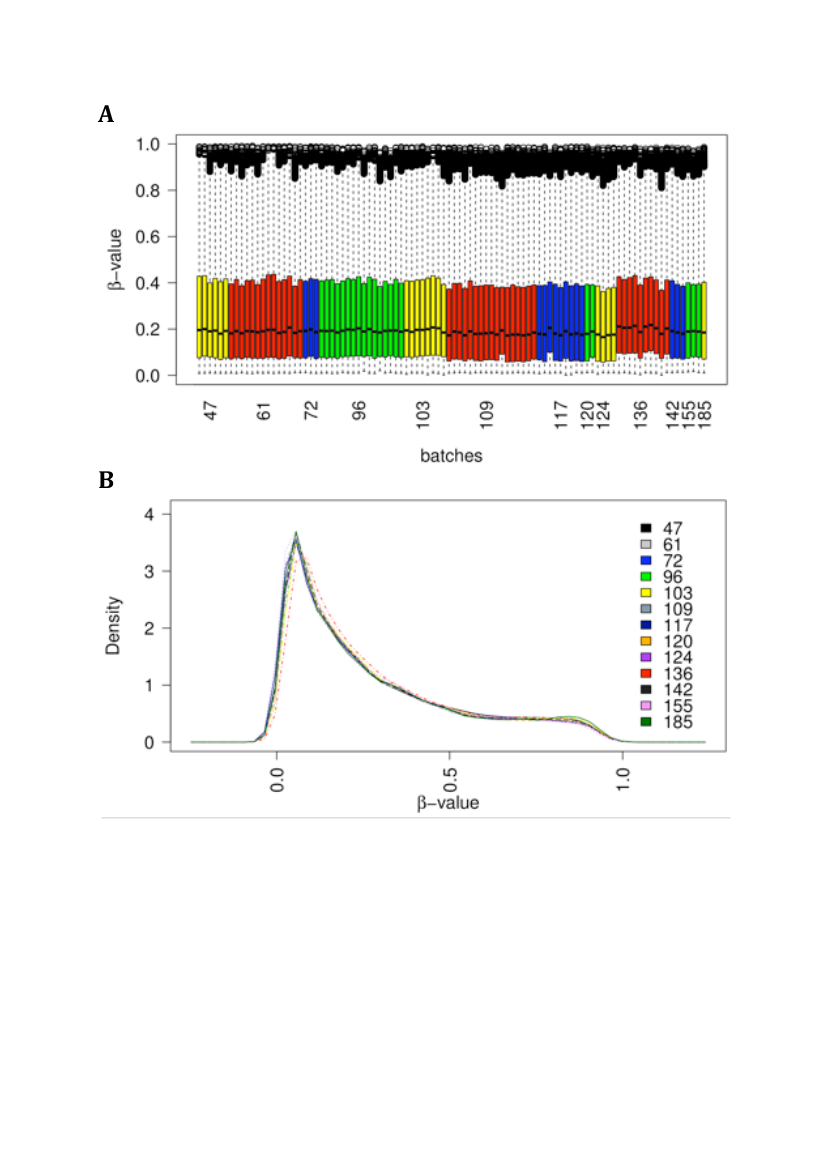

Supplement: S11 Fig — A. Per sample boxplot B. Density plot. Functional normalization was able to adjust the batch effect equally well as BEclear since S11A Fig looks very similar to what was obtained after BEclear correction (S6B Fig). (TIFF) [file pone.0159921.s011.tiff]

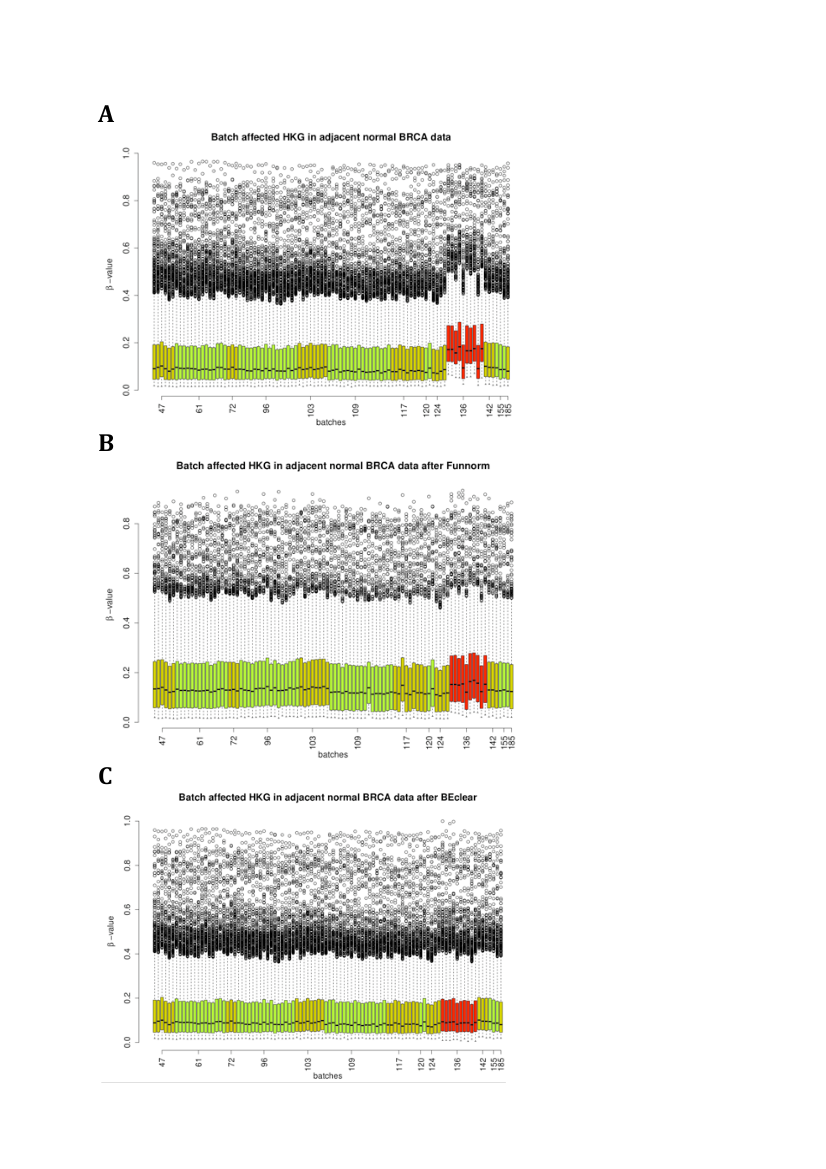

Supplement: S12 Fig — before any batch effect adjustment B. after functional normalization C. after batch effect correction with BEclear. The most affected batch is marked in red. (TIFF) [file pone.0159921.s012.tiff]

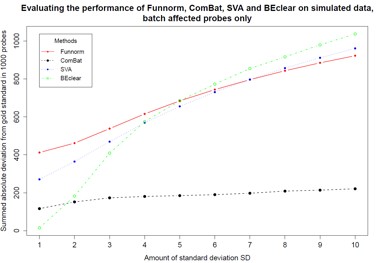

Supplement: S13 Fig — As a measure of performance we used the total absolute difference of the β-values between gold standard data and corrected entries for 1000 batch affected probes (out of 8000 probes). (PNG) [file pone.0159921.s013.png]

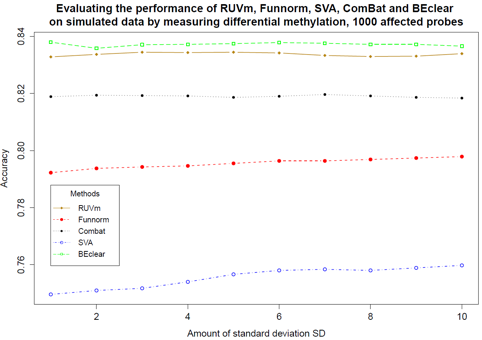

Supplement: S14 Fig — Here, only 1000 probes were perturbed instead of 4000). For all methods the list of differentially methylated genes (DMG) was obtained and then compared to the list of DMG for gold standard data. Here batch affect was introduced to 1000 probes (out of 8000). The x-axis indicates for the magnitude of the introduced batch effect. (PNG) [file pone.0159921.s014.png]
