# Supplementary material for: BEclear: Batch Effect Detection and Adjustment in DNA Methylation Data
Source: PLoS One. 2016 Aug 25;11(8):e0159921. doi: 10.1371/journal.pone.0159921 (PMC4999208; doi:10.1371/journal.pone.0159921)
Supplement: S1 Table — Cancer types and batches which were identified to have a batch effect are marked in bold. This table contains the description of cancer types, batch identifiers obtained from the TCGA portal and the batch effect score (see Eq (1) main text). Only those batches with BE-score over 0.01 are listed here since, generally, every batch has some extremely small non-zero BE-scores. This is due to some variation in a few genes and only in rare cases the BE-score for a batch is exactly zero. All the batches belonging to LUSC have a BE-score in the range of (0; 0.01) because not more than 97 genes in a single batch behave differently compared to other batches. The Dixon test yielded p-values for testing whether the BE-score of one of the batches differs significantly from the others in the same cancer type. Note that Dixon test is applied to a set of batches for one condition, not to a single batch. Hence, the reported p-value belongs to the respective set of batches. Note also that the Dixon test should not be considered alone but with BE-score threshold = 0.1, since it is prone to finding significant deviations of BE-scores if they are close to 0, as in the case for UCEC (adjacent normal samples) and THCA (adjacent normal and tumor samples) data. (DOCX) [file pone.0159921.s015.docx]

| **batch ID** | **BE score** | **Dixon test p-values** |
| --- | --- | --- |
| **Breast invasive carcinoma BRCA, adjacent normal data, represented by 13 batches** | | |
| 47 | 0.015 | < 0.001 |
| 61 | 0.038 |  |
| 109 | 0.013 |  |
| **136** | **0.605** |  |
| BRCA, tumor data, represented by 32 batches | | |
| 61 | 0.019 | < 0.001 |
| 80 | 0.022 |  |
| 124 | 0.017 |  |
| **136** | **0.188** |  |
| 322 | 0.017 |  |
| 334 | 0.053 |  |
| Uterine Corpus Endometrial Carcinoma UCEC, adjacent normal data, represented by 12 batches | | |
| 104 | 0.027 | < 0.001 |
| UCEC, tumor data, represented by 23 batches | | |
| 49 | 0.021 | 0.008785 |
| 92 | 0.036 |  |
| 156 | 0.015 |  |
| 186 | 0.017 |  |
| Thyroid carcinoma THCA, adjacent normal data, represented by 12 batches | | |
| 115 | 0.014 | < 0.001 |
| THCA, tumor data, represented by 17 batches | | |
| 115 | 0.016 | < 0.001 |
| Kidney renal clear cell carcinoma KIRC, adjacent normal data, represented by 5 batches | | |
| 82 | 0.042 | 0.01228 |
| **KIRC, tumor data, represented by 12 batches** | | |
| **32** | **0.185** | < 0.001 |
| 387 | 0.037 |  |
| Head and Neck squamous cell carcinoma HNSC, adjacent normal data, represented by 4 batches | | |
| 83 | 0.0108 | 0.1455 |
| 107 | 0.0106 |  |
| 151 | 0.0143 |  |
| HNSC, tumor data, represented by 18 batches | | |
| 107 | 0.013 | 0.01981 |
| 145 | 0.015 |  |
| 260 | 0.0107 |  |
| 265 | 0.032 |  |
| 403 | 0.019 |  |
| Lung adenocarcinoma LUAD, adjacent normal data, represented by 4 batches | | |
| 37 | 0.015 | 0.2292 |
| LUAD, tumor data, represented by 18 batches | | |
| 37 | 0.027 | 0.1738 |
| 52 | 0.016 |  |
| 84 | 0.02 |  |
| Lung squamous cell carcinoma LUSC represented by 4 batches of adjacent normal samples and 16 batches of tumor samples has no batches with BE-score >0.01 | | |

**Table S1.** BE scoring of DNA methylation data for 7 different cancer types from the TCGA portal. Cancer types and batches which were identified to have a batch effect are marked in bold. This table contains the description of cancer types, batch identifiers obtained from the TCGA portal and the batch effect score (see eq. (1) main text). Only those batches with BE-score over 0.01 are listed here since, generally, every batch has some extremely small non-zero BE-scores. This is due to some variation in a few genes and only in rare cases the BE-score for a batch is exactly zero. All the batches belonging to LUSC have a BE-score in the range of (0; 0.01) because not more than 97 genes in a single batch behave differently compared to other batches. The Dixon test yielded p-values for testing whether the BE-score of one of the batches differs significantly from the others in the same cancer type. Note that Dixon test is applied to a set of batches for one condition, not to a single batch. Hence, the reported p-value belongs to the respective set of batches. Note also that the Dixon test should not be considered alone but with BE-score threshold = 0.1, since it is prone to finding significant deviations of BE-scores if they are close to 0, as in the case for UCEC (adjacent normal samples) and THCA (adjacent normal and tumor samples) data.
