# Supplementary material for: BEclear: Batch Effect Detection and Adjustment in DNA Methylation Data
Source: PLoS One. 2016 Aug 25;11(8):e0159921. doi: 10.1371/journal.pone.0159921 (PMC4999208; doi:10.1371/journal.pone.0159921)
Supplement: S2 Table — The median difference counts the number of genes for which the median DNA methylation in this batch differs from its median in all other batches by a value falling into the respective intervals specified at the top. The BEscore is computed according to Eq (1) in the main text. (DOCX) [file pone.0159921.s016.docx]

| **batch ID** | **Median difference** | | | | | **BE-score** |
| --- | --- | --- | --- | --- | --- | --- |
|  | **[0.05;0.1)** | **[0.1;0.2)** | **[0.2;0.3)** | **[0.3;0.4)** | **[0.4;0.5)** |  |
| 47 | 91 | 32 | 4 | 0 | 0 | 0.015 |
| 61 | 274 | 63 | 8 | 0 | 0 | 0.039 |
| 72 | 6 | 5 | 1 | 0 | 0 | 0.002 |
| 96 | 33 | 2 | 0 | 0 | 0 | 0.003 |
| 103 | 13 | 0 | 0 | 0 | 0 | 0.001 |
| 109 | 143 | 5 | 0 | 0 | 0 | 0.014 |
| 117 | 93 | 3 | 0 | 0 | 0 | 0.009 |
| 120 | 3 | 0 | 0 | 0 | 0 | 0 |
| 124 | 14 | 1 | 0 | 0 | 0 | 0.001 |
| 136 | 3992 | 1159 | 104 | 9 | 1 | 0.605 |
| 142 | 10 | 0 | 1 | 0 | 0 | 0.001 |
| 155 | 8 | 0 | 1 | 0 | 0 | 0.001 |
| 185 | 0 | 0 | 0 | 0 | 0 | 0 |

**Table S2.** BE scoring of batches in BRCA adjacent normal data from TCGA. The median difference counts the number of genes for which the median DNA methylation in this batch differs from its median in all other batches by a value falling into the respective intervals specified at the top. The BEscore is computed according to equation (1) in the main text.
