# Supplementary material for: BEclear: Batch Effect Detection and Adjustment in DNA Methylation Data
Source: PLoS One. 2016 Aug 25;11(8):e0159921. doi: 10.1371/journal.pone.0159921 (PMC4999208; doi:10.1371/journal.pone.0159921)
Supplement: S3 Table — (DOCX) [file pone.0159921.s017.docx]

| **batch ID** | **Median difference** | | | | **BE-score** |
| --- | --- | --- | --- | --- | --- |
|  | **[0.05;0.1)** | **[0.1;0.2)** | **[0.2;0.3)** | **[0.3;0.4)** |  |
| 109 | 37 | 2 | 0 | 0 | 0.0037 |
| 117 | 25 | 3 | 0 | 0 | 0.0028 |
| 120 | 12 | 4 | 0 | 0 | 0.0018 |
| 124 | 166 | 13 | 1 | 0 | 0.0176 |
| 136 | 1661 | 199 | 10 | 1 | 0.1887 |
| 142 | 19 | 3 | 0 | 0 | 0.0022 |
| 147 | 14 | 2 | 0 | 0 | 0.0016 |
| 155 | 6 | 2 | 0 | 0 | 0.0009 |
| 167 | 13 | 1 | 0 | 0 | 0.0013 |
| 177 | 69 | 11 | 0 | 0 | 0.0082 |
| 185 | 1 | 2 | 0 | 0 | 0.0004 |
| 202 | 4 | 1 | 0 | 0 | 0.0005 |
| 216 | 1 | 0 | 0 | 0 | 0.0001 |
| 227 | 13 | 2 | 0 | 0 | 0.0015 |
| 234 | 3 | 1 | 0 | 0 | 0.0004 |
| 239 | 17 | 2 | 0 | 0 | 0.0019 |
| 255 | 12 | 2 | 0 | 0 | 0.0014 |
| 271 | 31 | 7 | 0 | 0 | 0.004 |
| 296 | 3 | 2 | 0 | 0 | 0.0006 |
| 305 | 12 | 3 | 0 | 0 | 0.0016 |
| 61 | 165 | 24 | 0 | 0 | 0.0191 |
| 322 | 102 | 31 | 5 | 2 | 0.0176 |
| 334 | 300 | 93 | 16 | 7 | 0.0531 |
| 338 | 16 | 2 | 0 | 0 | 0.0018 |
| 72 | 45 | 5 | 0 | 0 | 0.0049 |
| 74 | 79 | 10 | 0 | 0 | 0.0089 |
| 80 | 189 | 26 | 2 | 0 | 0.0223 |
| 93 | 0 | 0 | 0 | 0 | 0 |
| 96 | 18 | 0 | 0 | 0 | 0.0016 |
| 103 | 9 | 1 | 0 | 0 | 0.001 |
| 360 | 13 | 3 | 0 | 0 | 0.0017 |
| 372 | 24 | 6 | 1 | 0 | 0.0036 |

**Table S3.** BE scoring of batches in BRCA tumor data from TCGA (similar to table S1).
