# Supplementary material for: BEclear: Batch Effect Detection and Adjustment in DNA Methylation Data
Source: PLoS One. 2016 Aug 25;11(8):e0159921. doi: 10.1371/journal.pone.0159921 (PMC4999208; doi:10.1371/journal.pone.0159921)
Supplement: S4 Table — Listed are the number of highly co-methylated pairs of genes (Pearson correlation higher than 0.75 or lower than -0.75) for three different types of data after batch effect adjustment with BEclear and before. (DOCX) [file pone.0159921.s018.docx]

|  | **Tumor samples** | **Adjacent normal samples** | **Combined samples** |
| --- | --- | --- | --- |
| Number of co-methylated gene pairs in dataset before BE correction | 115 | 8206 | 9592 |
| Number of co-methylated gene pairs in dataset after BE correction | 112 | 4517 | 10616 |
| Number of common co-methylated gene pairs in BE-corrected and uncorrected datasets | 112 | 4228 | 8893 |
| Number of co-methylated gene pairs present in corrected dataset and absent in uncorrected | 0 | 289 | 1723 |
| Number of co-methylated gene pairs present in uncorrected dataset and absent in corrected | 3 | 3978 | 699 |

**Table S4.** Results of co-methylation analysis. Listed are the number of highly co-methylated pairs of genes (Pearson correlation higher than 0.75 or lower than -0.75) for three different types of data after batch effect adjustment with BEclear and before.
